# Risk Factors for Knee Osteoarthritis in Retired Professional Footballers: A Cross-Sectional Study

**DOI:** 10.1097/JSM.0000000000000742

**Published:** 2019-05-18

**Authors:** Sanjay M. Parekh, Gwen S. Fernandes, Jonathan P. Moses, Colin W. Fuller, Brigitte E. Scammell, Mark E. Batt, Weiya Zhang, Michael Doherty

**Affiliations:** * Division of Rheumatology, Orthopaedics and Dermatology, Academic Rheumatology, School of Medicine, University of Nottingham, Nottingham, United Kingdom;; † Arthritis Research UK Centre for Sport, Exercise and Osteoarthritis, Queens Medical Centre, Nottingham, United Kingdom;; ‡ Arthritis Research UK Pain Centre, Nottingham City Hospital, Nottingham, United Kingdom;; § Nottingham University Hospitals NHS Trust, Queen's Medical Centre, Nottingham, United Kingdom; and; ¶ Colin Fuller Consultancy Ltd, East Leake, United Kingdom.

**Keywords:** football, knee osteoarthritis, knee replacement, knee pain, risk factors

## Abstract

To determine risk factors for 3 knee osteoarthritis (KOA) outcomes, knee pain (KP), radiographic KOA (RKOA), and total knee replacement (TKR) in professional footballers.

**Design:**

This was a cross-sectional study involving a postal questionnaire, followed by radiographic assessment in a subcohort of responders.

**Settings and Participants:**

Four thousand seven hundred seventy-five questionnaires were sent to retired professional footballers, who had played in the English football league, and 1207 responded. Of these, 470 underwent knee radiographs.

**Assessment of Risk Factors:**

Potential factors include age, body mass index (BMI), knee alignment, a history of football-related knee injury, and training hours (during career) were collected through the questionnaire.

**Main Outcome Measures:**

Knee osteoarthritis outcomes were current KP (pain for most days of the previous month), TKR (self-reported), and RKOA (observed through radiographs).

**Results:**

Football-related injury was the strongest risk factor for KP [adjusted odds ratio (aOR), 4.22; 95% confidence interval (CI), 3.26-5.48], RKOA [aOR, 2.88; 95% CI, 1.81-4.59], and TKR [aOR, 4.83; 95% CI, 2.87-8.13]. Footballers had a 7% increased risk of RKOA for every 1000 hours trained. Although age and gout were associated with all 3 KOA outcomes, BMI, nodal osteoarthritis (OA), a family history of OA, knee malalignment, and 2D:4D ratio were associated with one or another of these 3 KOA outcomes.

**Conclusion:**

This study is the first to examine KOA risk factors in retired professional footballers. The study has identified several risk factors, both specific (eg, knee injury and training dose) and nonspecific (eg, age and gout) to footballers. This may be used to develop prevention strategies to reduce the risk of KOA in professional footballers after retirement.

## Abstract 2

Supplemental Digital Content is Available in the Text.

## INTRODUCTION

Osteoarthritis (OA) is a common complex disorder^1^ with multiple risk factors, including age, body mass index (BMI), previous injury, and occupation.^2^ A meta-analysis in 2011 has previously demonstrated a strong association between a history of knee injuries and subsequent knee OA (KOA) in the general population.^3^

Globally, more than 265 million people play football,^4^ and of these, 110,000 are professional male footballers.^5^ The sport is physically demanding, and players are at high risk of injury,^6^ especially during match play, and approximately 18% of all injuries sustained occur at the knee.^7,8^ Such injuries can lead to both short-term and long-term consequences, including OA,^9^ specifically KOA.^10^

Although footballers are perceived to be at greater risk of KOA compared with the general population, the literature is limited^11–14^ and inconclusive.^9,10^ Previous studies have been heterogeneous in design and lacked general population controls. The definition of KOA also varied, and sample sizes were relatively small. Footballers' professional status could not be ascertained from older studies.^15,16^ None of these studies formally examined the specific factors associated with KOA in professional footballers.

A recent study comparing retired professional male footballers with general population controls demonstrated an increased prevalence of knee pain (KP), radiographic KOA (RKOA), radiographic knee chondrocalcinosis (CC), and total knee replacement (TKR) in footballers.^12^ The aim of this study was to examine, within retired professional footballers, potential risk factors that may account for the increased prevalence of KP, RKOA, and TKR.

## METHODS

### Research Ethics Approval

This study was approved by the Nottingham University Hospital NHS Trust and the Nottingham Research Ethics (Ref: 14/EM/0045) and registered on the clinicaltrails.gov portal (NCT02098044). All participants gave implied consent by responding to the questionnaire survey and providing informed written consent before having knee radiographs.

### Study Design

This was a cross-sectional study involving a postal questionnaire distributed to retired professional footballers across the United Kingdom. A subcohort of footballers subsequently underwent bilateral knee radiographs at their nearest Spire Healthcare Hospital. Three independent nested case–control comparisons were performed; cases were defined as footballers with the outcome (namely, KP, RKOA, or TKR), and controls were defined as footballers without the outcome.

### Participants

Questionnaires were distributed to footballers through professional football clubs and their former players associations (top 4 tiers of the English Football League in the 2014/2015 season), the Professional Footballers' Association, and the League Managers Association. A convenience sample of eligible participants was recruited from male footballers, aged 40 years and older, who played professional football, and had responded to the questionnaire. Footballers who indicated a willingness to undergo knee radiographs, had not had bilateral TKR, and lived within 40 miles of a Spire Hospital were eligible for radiographic assessment. All questionnaire responses were verified (by name, date of birth, career duration, and matches played) to published records^17^ for responder integrity.

### Exposures

The questionnaire collected data on demographic, constitutional, and biomechanical risk factors, and career details of the footballers. These included age, BMI (obtained from height and weight), and a family history (first-degree relatives) of OA (in knees, hips, or hands) or joint replacement (knees or hips). Nodal OA, identified using a validated line-drawing instrument^18^ (through the questionnaire), was present if there were nodes (Heberden or Bouchard) on at least 2 digits of each hand. A pattern 3 2D:4D ratio (ring finger longer than index finger) and self-reported knee alignment in their 20s (reflecting constitutional alignment), as well as current alignment, were determined using validated line-drawing instruments (through the questionnaire).^19,20^ Other variables, obtained from the questionnaire, included current body pain, identified using a body pain line-drawing mannequin,^21,22^ any comorbidities and current medications, and high-risk occupations (eg, coal miners or carpet layers) after retirement from professional football, and were identified according to pre-existing literature.^23–27^

Footballers were asked whether they had sustained a significant knee injury over the course of their football career, and how many injuries they had sustained. We defined a significant knee injury as “one which caused you pain for most days of at least a 3-month period and resulted in an absence from all training and matches during this time.” We wanted to explore the relationship with more serious injuries to the knee, and after feedback from a pilot survey (distributed to former footballers), a period of 3 months was suggested. Such injuries may include ligament and meniscus injuries, but as injuries were self-reported rather than confirmed through medical records, we felt it prudent to explore the type of injury.

They were also asked about their career duration, number of matches, and level played at, average weekly training duration, and footedness. Career training dose was calculated by multiplying the number of hours played per week by 40 weeks (average season) by career duration.

### Outcome Measures

Knee OA outcomes were current KP (assessed by indication on the body pain mannequin of “any pain for most days of the previous month” in or around their knees), TKR (self-reported in the questionnaire), and RKOA (confirmed by radiography).

Radiographs were standardized as a bilateral weight-bearing, semiflexed posterior–anterior tibiofemoral view (using the Rosen template to standardize degree of knee flexion and rotation)^28^ and individual 30-degree flexion skyline patellofemoral (PF) views (using a jig). RKOA was measured using the Nottingham Line Drawing Atlas (NLDA) and defined as present if there was “definite osteophytes (score ≥ 2) in any compartment and joint space narrowing (JSN) (score ≥ 2) in at least one of the tibiofemoral (TF) or PF compartments.”

Radiographic CC in either hyaline or fibrocartilage was also assessed and defined as present or absent. All radiographs were scored by a single assessor (G.S.F.), and a small sample (21 participants; 40 knees) was used to determine intrarater agreement using the NLDA (Kappa 1.00).

### Statistical Analysis

For the sample size calculation, it was estimated that the source population for this study is 18,500 based on an estimate of 50,000 footballers and a response rate of 37%.^29^ Assuming the prevalence of KOA was 30%^30^ and an acceptable deviation was 3%, the sample size required was 855 footballers, with a power of 95% and a significance level of *P* = 0.05.

Prevalence of KOA was calculated overall and by age and BMI. Categorical variables were reported as frequencies and percentages and continuous variables as mean values and SDs. Comparisons were made between footballers with and without each KOA outcome, and the risk factors associated were analyzed with 1 logistic regression model for each factor to estimate the odds ratio (OR) and 95% confidence intervals (CIs). Logistic regression was used to adjust for confounding factors (age and BMI) and to determine the relationship between number of injuries and outcomes.

Receiver operator characteristic (ROC) analysis determined the contribution of significant risk factors.^31^ This was calculated as the area under the curve (AUC, scale 0-1) for the full model (all factors) and the partial model(s). The proportional risk contribution was calculated by (AUC_full_ − AUC_partial_)/(AUC_full_ − 0.5); where AUC_full_ is the AUC for the full risk model, AUC_partial_ is the AUC for the partial risk model without the specific risk factor, and 0.5 is the AUC under the diagonal line of the ROC curve that reflects no discrimination or prediction of the specific risk factor(s) for the outcome of interest.

## RESULTS

### Recruitment

**Figure 1.**
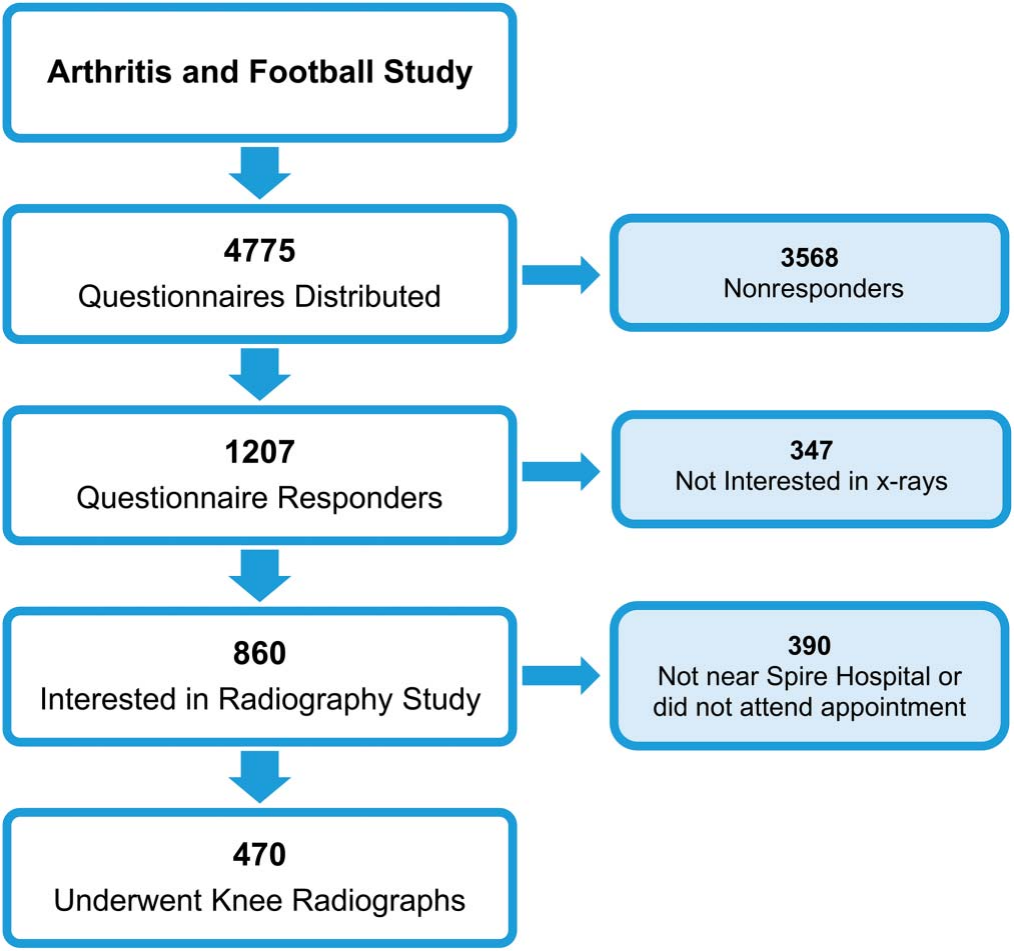
Recruitment of participants (footballers). This figure shows the number of retired professional footballers recruited for both the questionnaire phase and radiographic phase of the study. Footballers who did not respond or were ineligible for the x-ray phase of the study were excluded.

Four thousand seven hundred seventy-five questionnaires were mailed to footballers through professional football clubs and their former players associations (Figure 1). One thousand two hundred seven returned their completed questionnaires (25.3%), and of these, 470 footballers underwent radiographic assessment.

### Characteristics of Population

Table 1 reports the constitutional, local, and biomechanical and football-specific factors of the footballers who responded to the questionnaire. The average age and BMI were 59.0 years (SD ± 11.7) and 27.3 kg/m^2^ (SD ± 3.2), respectively. Almost two-thirds had a pattern 3 2D:4D ratio and less than 10% had nodal OA. The prevalence of familial OA in the knee (19.6%), hand (15.9%), or hip (14.0%) was similar.

**TABLE 1.** Baseline Characteristics and Prevalence

	Study Population (n = 1207)
Age (yr), mean (SD)	59.0 (11.7)
BMI (kg/m^2^), n (%)	
Normal	255 (21.6)
Overweight	726 (61.5)
Obese	200 (16.9)
Digit (2D:4D) ratio, n (%)	733 (60.7)
Nodal OA, n (%)	86 (7.1)
Family history of OA, n (%)*	377 (31.2)
Suffers from gout, n (%)	142 (11.8)
Constitutional malalignment, n (%)†	193 (16.0)
High-risk occupation (after football), n (%)	742 (61.5)
Knee injury, n (%)	778 (64.5)
Career training dose (hours), mean (SD)‡	7840 (4180)
Matches played, mean (SD)§	470 (240)
Main tier, n (%)¶	
Tier 1	354 (29.3)
Tier 2	253 (21.0)
Tier 3	223 (18.5)
Tier 4	119 (9.9)
Other tiers║	207 (17.1)
Position, n (%)	
Goalkeeper	113 (9.4)
Defender	404 (33.5)
Midfielder	336 (27.8)
Forward	351 (29.1)
Outcome measures (self-reported), n (%)	
Current KP	630/1207 (52.2)
TKR	134/1207 (11.1)
Physician-diagnosed KOA	341/1207 (28.3)
Outcome measures (radiographic), n (%)	
RKOA	301/470 (64.0)
CC	114/470 (24.3)

The first part of this table reports the baseline characteristics for the study population, which was 1207 footballers, who responded to the questionnaire survey. The prevalence of the outcome measures, both self-reported (questionnaire) and radiographic, is reported in the second part of this table.

* Family history includes first-degree relatives (mother, father, or sibling) with a history of knee, hip, hand OA or who have undergone a knee replacement.

† Constitutional malalignment is a self-reported malalignment measured in the footballers 20s (reflective of natural alignment).

‡ Career training dose is the cumulative hours a footballer spent in training practice calculated based on training duration (hours per wk) X weeks per season (40) X career duration (presented to 3 significant figures).

§ (Presented to 2 significant figures).

¶ Main tier is the tier (league) footballers played in for most of their career.

║ Other tiers include reserve leagues, lower leagues, and other countries (leagues not part of the English Football League system).

A total of 16.0% of footballers reported constitutional varus or valgus alignment, but this increased to 24.6% for current alignment. Almost 65% of footballers reported at least 1 significant football-related knee injury during their career. A total of 38.4% of footballers retired from the game because of a significant football-related injury of which more than half (52.5%) were due to significant knee injuries. On average, footballers reported training for 3 hours 5 days a week, equating to a mean of almost 8000 hours over the course of their career (average duration of 14 years). Footballers played an average of 450 matches, yet 10% had played over 800 matches.

### Prevalence

Table 1 also presents the prevalence of the outcome measures in retired footballers. A total of 52.2% had KP, 64.0% had RKOA, and 11.1% had undergone a TKR. The prevalence of each outcome measure increased by age (see **Appendix 1A**, **Supplemental Digital Content 1**, http://links.lww.com/JSM/A207). Knee pain prevalence rose slightly up to the age of 60 years but declined slightly thereafter. Both the prevalence of RKOA and TKR increased with age. For footballers older than 80 years, 41% had KP, 85.7% had RKOA, and 33.3% had undergone a TKR. KP and TKR showed successive increases in prevalence from normal weight to overweight and obese (see **Appendix 1B**, **Supplemental Digital Content 1**, http://links.lww.com/JSM/A207). Prevalence of RKOA was highest (71%) in those who were obese (BMI ≥ 30 kg/m^2^).

### Risk Factors

Table 2 shows the association between potential risk factors and each outcome, after adjustment for age and BMI. Sustaining at least 1 football-related knee injury conferred the greatest odds of KP [adjusted OR (aOR), 4.22; 95% CI, 3.26-5.48], RKOA [aOR, 2.88; 95% CI, 1.81-4.59], and TKR [aOR, 4.83; 95% CI, 2.87-8.13].

**TABLE 2.** Adjusted Odds Ratio for Primary Outcome Risk Factors

Exposure	Adjusted Odds Ratio (95% CI)*		
	KP	RKOA	TKR
Age (yr)	0.99 (0.98-1.00)†	1.08 (1.05-1.10)†	1.09 (1.07-1.11)†
BMI (kg/m^2^)			
Normal	Reference	Reference	Reference
Overweight	1.50 (1.13-2.01)†	0.92 (0.55-1.54)	1.50 (0.90-2.50)
Obese	2.28 (1.55-3.33)†	1.18 (0.59-2.36)	2.03 (1.11-3.73)‡
Pattern 3 digit ratio	1.34 (1.05-1.72)‡	0.92 (0.59-1.44)	1.33 (0.86-2.05)
Nodal OA	1.21 (0.77-1.92)	0.81 (0.29-2.30)	2.24 (1.28-3.90)†
Familial OA	1.51 (1.17-1.94)†	1.49 (0.99-2.26)	1.16 (0.76-1.77)
Suffers from gout	2.16 (1.46-3.20)†	2.71 (1.13-6.49)‡	1.76 (1.09-2.86)‡
Constitutional malalignment	1.28 (0.93-1.76)	1.15 (0.66-1.99)	2.04 (1.25-3.33)†
High-risk occupation	1.19 (0.94-1.51)	1.00 (0.66-1.51)	0.90 (0.61-1.33)
Knee injury	4.22 (3.26-5.48)†	2.88 (1.81-4.59)†	4.83 (2.87-8.13)†
Career training dose (per 1000 h)	1.00 (0.97-1.03)	1.07 (1.01-1.12)‡	0.98 (0.95-1.05)
Matches played (per 100 matches)	0.96 (0.91-1.00)	1.06 (0.97-1.15)	0.91 (0.83-0.99)‡
Main tier§			
One	0.97 (0.75-1.25)	0.81 (0.52-1.27)	1.11 (0.74-1.67)
Two	1.07 (0.80-1.42)	1.10 (0.86-1.40)	1.03 (0.64-1.67)
Three	1.09 (0.81-1.47)	1.05 (0.89-1.25)	0.91 (0.55-1.53)
Four	0.88 (0.60-1.30)	0.96 (0.82-1.12)	0.72 (0.32-1.63)
Position¶			
Goalkeeper	1.33 (0.89-1.98)	1.14 (0.58-2.24)	0.87 (0.45-1.68)
Defender	0.86 (0.68-1.11)	1.12 (0.90-1.39)	1.27 (0.84-1.92)
Midfielder	1.00 (0.77-1.29)	1.07 (0.91-1.24)	0.78 (0.49-1.23)
Forward	1.05 (0.81-1.35)	0.88 (0.78-0.98)‡	1.03 (0.69-1.56)

This table reports the aORs (adjusted for age and BMI), and 95% CIs of all potential risk factors for all 3 KOA outcomes, namely KP, RKOA, and TKR.

* Odds ratios adjusted for age and BMI.

† *P* < 0.01.

‡ *P* < 0.05.

§ Adjusted OR presented with respect to all other tiers.

¶ Adjusted OR presented with respect to all other positions.

**Figure 2.**
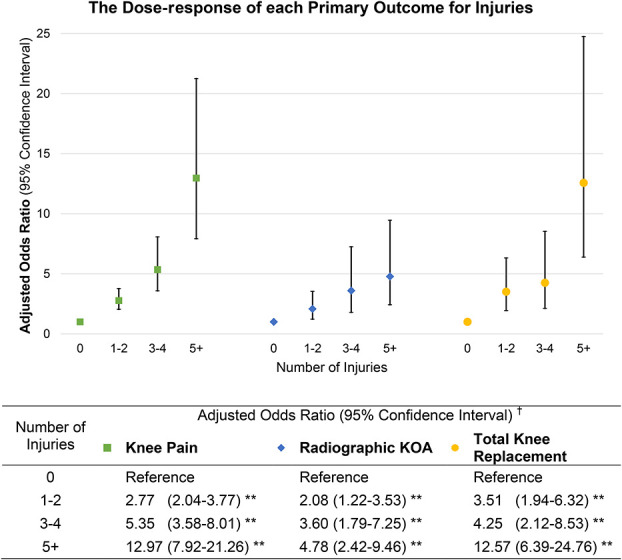
Dose response (OR) for primary outcomes for number of injuries. This figure presents the aOR for the risk of having outcomes of KOA, namely KP (green square), RKOA (blue diamond), and TKR (yellow circle), with increasing number of injuries. Data for the aOR and 95% CIs are included below the graph for reference. †OR adjusted for age and BMI; **P < 0.01.

The relationship between the number of injuries and OR of each outcome is shown in Figure 2. Footballers who had sustained 1 or 2 knee injuries had significant increased odds of KP [aOR, 2.77; 95% CI, 2.04-3.77], RKOA [aOR, 2.08; 95% CI, 1.22-3.53], and TKR [aOR, 3.51; 95% CI, 1.94-6.32]. The odds increased to almost 13 times greater for KP [aOR, 12.97; 95% CI, 7.92-21.26] and TKR [aOR, 12.57; 95% CI, 6.39-27.76] and 5 times greater for RKOA [aOR, 4.78; 95% CI, 2.42-9.46] in footballers who suffered at least 5 or more significant knee injuries.

Footballers who reported having gout also had significantly greater odds of each outcome. Age was significantly associated with increased odds of RKOA and TKR only, whereas having a pattern 3 2D:4D ratio and a family history of OA were associated with KP only. Footballers had 7% increased odds of RKOA for every 1000 hours trained [aOR, 1.07; 95% CI, 1.01-1.12], but 9% reduced odds of TKR [aOR, 0.91; 95% CI, 0.83-0.99] for every 100 matches they played.

### Receiver Operator Characteristic Analysis

The ROC AUC values for full and partial risk models are reported in Table 3. The ROC analysis shows that the identified factors in the full model explain 73%, 84%, and 85% of the risk of having KP, RKOA, and TKR respectively. Of the full AUC, injury contributed 40.2% of the risk for KP, but only 6.7% of the risk for RKOA and 9.9% of the risk for TKR. Age, however, contributed the greatest risk for RKOA (44.6%) and TKR (26.1%). **Supplemental Digital Content 2** (see **Appendix 2**, http://links.lww.com/JSM/A208) shows the corresponding ROC AUC for the full model, the model with injury removed, and the model with all significant risk factors removed.

**TABLE 3.** Receiver Operator Characteristic Area Under the Curve (AUC) and Proportional Risk Contribution (PRC)

	AUC	95% CI	% PRC
Knee pain (n = 1207)			
Full model	0.7317	0.7004-0.7630	
Partial model without			
Knee injury	0.6385	0.6042-0.6727	40.22
Gout	0.7293	0.6978-0.7608	1.04
Digit (2D:4D) ratio	0.7259	0.6945-0.7573	2.50
Familial OA	0.7268	0.6953-0.7583	2.11
High-risk occupation	0.7286	0.6971-0.7602	1.34
BMI	0.7203	0.6885-0.7521	4.92
Partial model without all significant factors (listed above)	0.5645	0.5289-0.6000	72.16
Radiographic knee OA (n = 470)			
Full model	0.8424	0.7616-0.9232	
Partial model without			
Knee injury	0.8195	0.7330-0.9060	6.69
Age	0.6896	0.5842-0.7950	44.63
Position	0.8242	0.7399-0.9115	5.32
Partial model without all significant factors (listed above)	0.6320	0.5228-0.7412	61.45
Total knee replacement (n = 1207)			
Full model	0.8457	0.8056-0.8858	
Partial model without			
Knee injury	0.8115	0.7667-0.8564	9.89
Nodal OA	0.8403	0.7998-0.8808	1.56
Age	0.7554	0.7032-0.8076	26.12
BMI	0.8434	0.8033-0.8836	0.67
Matches played	0.8415	0.8018-0.8811	1.21
Partial model without all significant factors (listed above)	0.6450	0.5856-0.7044	58.06

This table presents the AUC with 95% CI, and corresponding PRC (%) for the full and partial models for each KOA outcome, namely KP, RKOA, and KPR. **Supplemental Digital Content 2** (see **Appendix 2**, http://links.lww.com/JSM/A208) presents the graphical output for the full and partial models. The full model includes all variables listed below:

Age + BMI + digit (2D:4D) ratio + nodal OA + familial OA (knee OA, hip OA, hand OA; and knee replacement) + gout + constitutional malalignment + high-risk occupation + knee injury + career training dose + matches played + main tier (played in) + position.

## DISCUSSION

This is the first comprehensive study to examine risk factors for KOA outcomes in retired professional footballers. Following our recent comparative study, which demonstrated that retired professional footballers had a higher prevalence of KOA than general population controls,^12^ this within-group case–control study identified that (1) knee injury is associated with KP, RKOA, and TKR; (2) there was a 7% increased odds for RKOA for every 1000 hours of training; and (3) age and gout are another 2 risk factors associated with all 3 KOA outcomes. Other risk factors, such as BMI, 2D:4D ratio and family history of OA vary between KOA outcomes.

The average age of footballers responding to this study was at least 3 years older than in other studies,^11,13,14,29,30^ and the majority were overweight or obese. The prevalence of knee injuries was far greater than previously reported,^29^ and outcomes were measured independently to account for the discordance between patient-centered outcomes (KP and TKR)^32^ and the presence of structural OA (RKOA). The prevalence of KP in footballers was far greater than previously reported.^30^ Although we use a specific definition of KP “*pain in or around the knee for most days of the previous month*,” previous studies' analysis of pain was more subjective, including the use of a visual analogue scale.^30^

The prevalence of RKOA was consistent with 2 previous studies^11,30^ but not another.^13^ However, these studies used the composite Kellgren Lawrence verbal descriptors, whereas in this study, we used the NLDA.^33,34^ The strengths of the NLDA are (1) the extent of JSN and osteophyte formation are independently determined using an interval scale; (2) structural change in both the TF and PF compartments are measured separately; and (3) JSN differences for men and women are presented in separate atlases. Another KOA outcome, TKR, is a surrogate for severe end-stage KOA and has not been previously assessed in the literature pertaining to ex-footballers.^9,10^ Radiographic CC was seen at a higher prevalence^12^ than expected in the normal male population.^35^ Deposition of both calcium phosphate and calcium pyrophosphate crystals is known to strongly associate both with OA^36^ and with previous joint trauma,^37^ but this element of OA and joint trauma has not been commented on previously in studies of ex-footballers.

Constitutional risk factors identified for KOA included increasing age, being overweight or obese, having a family history of OA, and having nodal OA, all of which are recognized risk factors.^2,38^ The high prevalence of pattern 3 2D:4D ratio, which has been associated with athletic ability as well as with KOA, is of interest in suggesting that elite footballers may genetically be at a higher odds of KOA. Gout also was associated with all KOA outcomes. Changes in OA cartilage encourage urate and calcium crystal formation, and equally, such crystal deposits can cause further mechanical and inflammatory damage to the joint in an amplification loop relationship.^39,40^ These associations re-emphasize that KOA is a common complex disorder.

Sustaining a knee injury, sufficient to require at least 3 months' time lost from playing football, was strongly associated with KOA in this study, and subsequent injuries further increased the odds. Significant direct injury causes structural and biomechanical insult and is a well-recognized risk factor for OA at many joint sites.^3^ Footballers are exposed to a high risk of injury,^6^ and the number and management of such injuries may result in irreversible damage. However, age, and not injury, is the greatest risk factor for both RKOA and TKR, which accords with evidence from population-based cohort studies that demonstrate age as a significant risk factor.^41,42^

Professional footballers in this study had a career duration of 14 years, which is longer than previously reported.^9,10^ A longer career duration meant footballers played a greater number of matches, but they had a reduced odds of having a TKR for every 100 matches they played. This suggests a potential “survival of the fittest” mechanism,^10^ whereby footballers who did not sustain injuries could stay in professional football for longer and were less likely to suffer negative longer-term consequences. However, prolonged exposure to training did result in footballers having an increased odds of RKOA for every 1000 hours trained. In addition to clinically overt acute injury, long-term exposure to football may create biomechanical stress on lower-limb joints through typical running, twisting, tackling, and jumping football-related activities.^43^ Such chronic repetitive microtrauma may not be clinically apparent or limit a footballer's ability to train or compete in match play but may increase the development of KOA. This phenomenon may also explain the underlying risk of KOA outcomes in footballers even after accounting for overt knee injury.

There are several limitations to this study. First, the questionnaire survey used to assess exposures and self-reported KOA outcomes may be prone to both recall and responder bias. Footballers who suffered significant injuries or KOA may better remember exposures and be more likely to return the questionnaire and more willing to undergo radiographic assessment, which may contribute to the high prevalence of KOA outcomes.

Second, despite this being the largest study of KOA in ex-professional footballers to date, there was a low response rate compared with all other previous studies.^14,29^

Furthermore, this study may have been prone to misclassification bias. Knee pain was defined as current, which may not fully cover those with chronic KP but who happened to have less pain or no pain in the past month. RKOA was confirmed using plain radiographs, which although cost effective compared with magnetic resonance imaging are relatively insensitive and unable to characterize soft-tissue abnormalities and intraosseous bone marrow lesions associated with KOA.^44,45^ Finally, only 73% to 85% of risk of the 3 KOA outcomes may be explained by the risk factors in this study (Table 3). This suggests there may be other factors that contribute to the risk of KOA but are not included in this study. However, that up to 85% of risk can be explained by a single study means that the major risk factors have been included.

The risk of KOA has long been believed to be greater within professional footballers than the general population, and this is the first study to highlight the magnitude and extent to which knee injury results in KOA. This would impact upon the quality of life and mental well-being of footballers who thus suffer from the chronic condition. Football organizations should be mindful of these data^46,47^ and should ensure that optimal conservative management and optimal recovery time after injury are allowed before footballers can return to play.^48^ An emphasis on effective management of modifiable risk factors, including injury, obesity, and chronic comorbidities (such as gout), is also imperative in the ex-footballer population. These measures could have both short-term (reduced time loss during players' careers) and longer-term (reduced risk of chronic musculoskeletal conditions) benefits.
